# Nivolumab Induces Durable Remission in Relapsed/Refractory ALK‐Negative ALCL After Allogeneic Blood Stem Cell Transplantation—A Case Report and Literature Review

**DOI:** 10.1002/jha2.70041

**Published:** 2025-06-04

**Authors:** Ben‐Niklas Baermann, Kathrin Nachtkamp, Paul Jäger, Paula Kessler, Emil Novruzov, Frederik L. Giesel, Helena Peters, Maximilian Seidl, Mark Uhlenbruch, Sascha Dietrich, Guido Kobbe

**Affiliations:** ^1^ Department of Hematology Oncology and Clinical Immunology Medical Faculty and University Hospital Duesseldorf, Heinrich‐Heine‐University Duesseldorf Düsseldorf Germany; ^2^ Center for Integrated Oncology Aachen‐Bonn‐Cologne‐Düsseldorf Düsseldorf Germany; ^3^ Department of Nuclear Medicine Medical Faculty and University Hospital Duesseldorf, Heinrich‐Heine‐University Duesseldorf Düsseldorf Germany; ^4^ Department of Diagnostic and Interventional Radiology Medical Faculty and University Hospital Duesseldorf, Heinrich‐Heine‐University Duesseldorf Düsseldorf Germany; ^5^ Institute of Pathology University Hospital Düsseldorf Düsseldorf Germany; ^6^ Department of Pneumology Cardiology and Intensive Care Medicine Florence‐Nightingale Hospital Düsseldorf Germany

**Keywords:** case report, GVHD, stem cell transplantation, T‐cell lymphoma

## Abstract

**Background:**

Relapsed or refractory anaplastic large cell lymphoma (r/r‐ALCL) remains an important therapeutic challenge, especially after allogeneic stem cell transplantation (alloHSCT). Due to encouraging results in other lymphoid diseases, PD‐1 blockade was investigated in T‐cell lymphoma in a small number of patients, but short duration of remission and significant adverse events were observed.

**Case Report:**

Here, we report successful and durable remission induction for more than 2 years with nivolumab for r/r‐ALCL even without any evidence of PD‐L1 expression.

**Conclusion:**

This report and literature review emphasizes the importance of the graft‐versus‐lymphoma effect and its potential pharmacological stimulators in the field of lymphoma treatment.

## 1 Introduction

While intensive chemoimmunotherapy results in 3‐year overall survival rates of 90% in anaplastic lymphoma kinase (ALK)‐positive anaplastic large cell lymphoma (ALCL), the outcome of ALK‐negative ALCL remains poor [1].

Brentuximab vedotin, a CD30‐antibody, offered a major improvement for remission induction at first line as well as in the treatment of patients with relapsed/refractory disease, especially in combination with consolidating autologous or allogeneic stem cell transplantation [2].

Since the rapid development of immune checkpoint blockade in Hodgkin lymphoma, efforts have been made to assess the efficacy of PD1‐antibodies like nivolumab in other lymphoma subtypes [3, 4].

In a Phase 2 trial, nivolumab has been tested in 12 patients with relapsed/refractory peripheral T‐cell lymphoma (PTCL). An overall response ratio of 33% was observed, with a median DOR of 3.6 months. Moreover, immune activation with hyperprogression was observed. These discouraging results might have been the reason not to support further investigation in larger cohorts [3].

## 2 Clinical Case

We here report a case of durable response to checkpoint inhibition in a patient with refractory ALCL after alloHSCT. A 58‐year‐old man received the diagnosis of classical Hodgkin's disease (nodular sclerosing type), Stage IVB with bone marrow infiltration in June 2017. He was treated with six cycles of escalated BEACOPP until December 2017 and achieved complete remission. In August 2018, new cervical, hilar, axillar, para‐aortal, inguinal, and iliacal lymph node swelling developed, and the histology showed CD30^+^ ALK‐negative ALCL. Staging revealed Stage IVB, including liver and spleen infiltration, and re‐examination of the original specimens from 2017 by a reference pathologist showed that also the 2017 original diagnosis of Hodgkin's disease had to be corrected to CD30^+^ ALK‐negative ALCL.

For the treatment of ALCL relapse, the patient received two cycles of DHAP without response and developed renal failure. Therapy was changed to brentuximab vedotin, which was stopped after two cycles because of no response. Following the application of two cycles of high‐dose cytarabine and mitoxantrone (HAM), the patient achieved a partial remission in December 2018, which only lasted until February 2019, when lymphonodal progression was treated with everolimus and radiotherapy. Again the patient achieved a partial remission.

In May 2019, he received an alloHSCT from a single‐antigen, mismatched, unrelated donor after conditioning with fludarabine, TBI 8 Gy, and post‐transplant cyclophosphamide. Consecutive [¹⁸F]fluorodeoxyglucose ([^18^F]FDG) positron emission tomography/computed tomography PET/CT in July 2019 and September 2019 showed complete metabolic remission of the lymphoma. Acute graft‐versus‐host disease (GvHD), as well as severe infections, steroid‐induced diabetes mellitus, acute renal failure, and NSTEMI marked the first year after transplantation.

Because of ongoing cytopenia, a CD34^+^ selected backup was given in January 2020.

In December 2020, [^18^F]FDG imaging revealed a disseminated disease progression involving various nodal and extranodal manifestations such as pleura, soft tissue or bone. Soft tissue biopsy showed a diffuse infiltration of CD30^+^, ALK‐negative ALCL with Ki67 proliferation index of more than 95%, and 5%–10% PD‐L1‐positive macrophages. Tumor cells did not express PD‐L1. Re‐exposition with brentuximab vedotin in December 2020 did not result in a significant reduction of the lymphoma mass.

**FIGURE 1:**
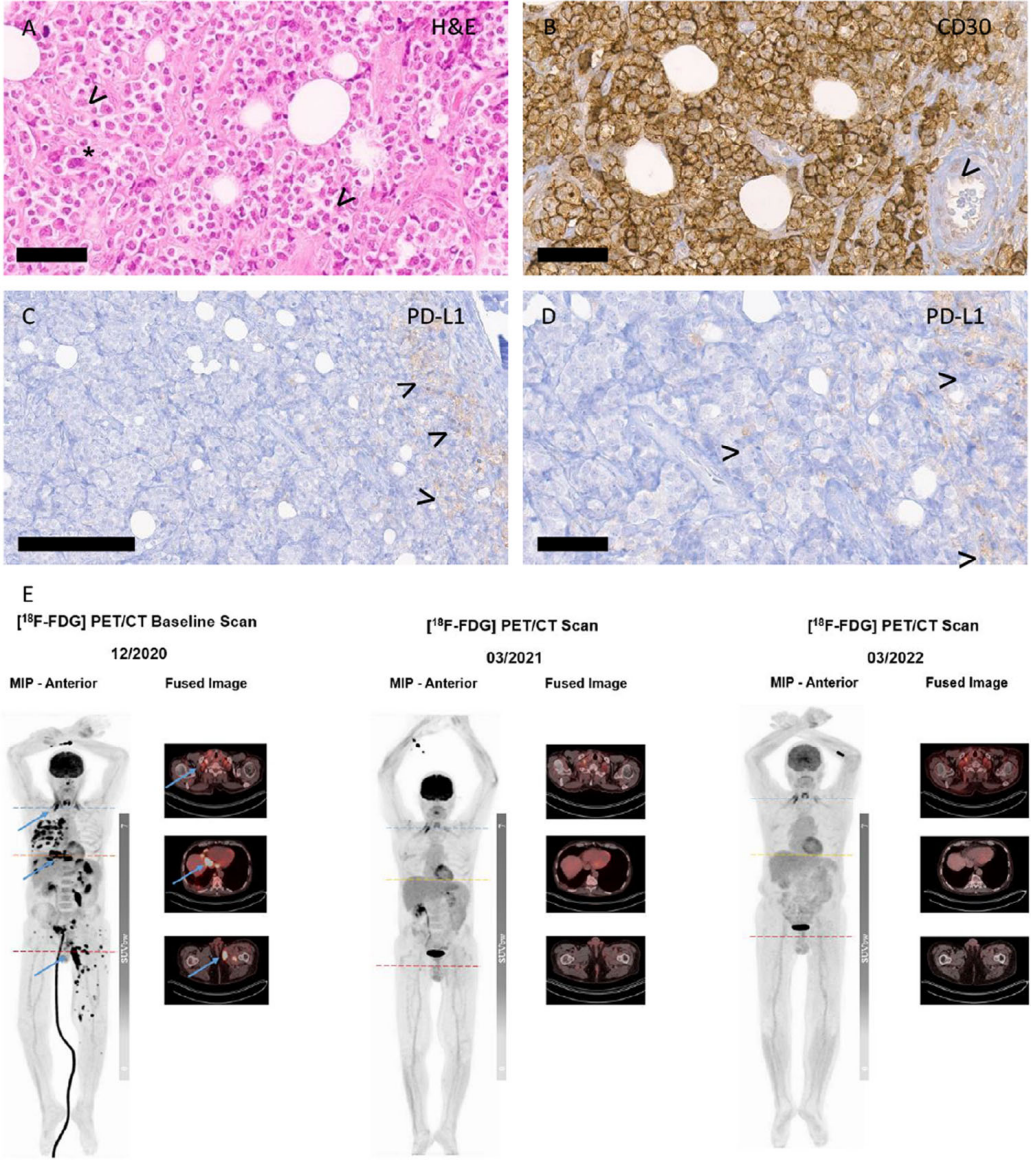
Pathologic features and therapy response to nivolumab. (A–D) Histology of ALCL. (A) Dense infiltrate of large and discohesive lymphoma cells with multiple mitoses (arrowheads, exemplary) and some very large, irregular nuclei (asterisk, exemplary). (B) Strong membranous CD30 positivity (DAB‐based brown coloration) on the lymphoma cells. Exemplary erythrocyte inside a small blood vessel marked by an arrowhead to visualize the large lymphoma cells. (C) PD‐L1 staining (Clone 28‐8) displaying negative lymphoma cells (left side) and a few positive macrophages at the infiltration margin (highlighted by arrowheads). (D) Higher magnification of (C), exemplary macrophages marked by arrowhead. All slides were digitized with an Aperio slide scanner (Leica, Nussloch, Germany), with a 40× objective and an original resolution of 0.253 µm per pixel. Magnifications were 40× for (A), (B), and (D), and 20× for (C) and indicated by bar (60 µm for A, B, D, and 200 µm for C). (E) This figure depicts the therapy response of immunotherapy with nivolumab on subsequent total body [^18^F]FDG PET/CT scans: [^18^F]FDG imaging following the initiation of nivolumab therapy demonstrated a rapid, excellent therapy response, with a complete resolution of all metabolically active manifestations on the baseline scan. The moderate [^18^F‐FDG] uptake in the cervical region on the subsequent scans points to a great extent symmetrical physiological glucose metabolism of the scalene muscles. Eventually, the metabolic therapy response is consistent with Deauville score of 2 according to Lugano classification.

Therefore, PD‐1 blockade was performed with nivolumab 240 mg starting in December 2020 and infused every 2 weeks. The patient received a total of 22 doses of nivolumab until December 2021. The interim [^18^F]FDG imaging after initiation of nivolumab therapy in March 2021 demonstrated an overwhelming metabolic response, given the high tumor burden at the baseline scan, and 3 months later in March 2022, the final response assessment using [^18^F]FDG PET/CT imaging confirmed a complete metabolic remission (Figure 1E).

The main reason for the discontinuation of nivolumab was the development of heart failure (NYHA III–IV) in December 2021 with a reduced ejection fraction of 26%. Myocardial magnetic resonance tomography revealed an inflammatory component, although myocardial biopsy did not reveal inflammatory lymphoid infiltration. Nevertheless, due to high clinical and radiological correlation highly suggestive of a nivolumab adverse reaction, high‐dose steroid therapy was initiated and resulted in symptomatic and ejection fraction improvement to greater than 35%.

After reduction of steroid therapy, severe acute cutaneous GvHD (Grade 3) was observed, which was successfully treated with increasing steroid doses. Finally, steroids could be tapered completely, and the patient remained free of further adverse immune reactions. Until January 2024, there has been no evidence of disease relapse without any other anti‐lymphoma treatment so far. The patient remains in good health with complete donor chimerism without evidence of chronic GvHD.

## 3 Discussion

To our knowledge, we report the first patient with ALK‐negative ALCL responding to nivolumab following relapse after alloHSCT, although tumor cells clearly did not express PD‐L1. The complete response now is ongoing for a least 34 months.

The potential of pembrolizumab in peripheral T‐cell lymphoma (two of 21 patients with ALK‐negative ALCL) was already assessed as consolidation after induction and autologous stem cell transplantation (autoHSCT). In this Phase 2 trial, no significant differences in the safety profile were observed, although the first infusion should have been given at least at Day 60 after autoHSCT [5].

Chan et al. reported a 35‐year‐old female patient with ALK‐negative ALCL relapse 3 months after mismatched alloHSCT. Intensive chemoimmunotherapy, autologous stem cell transplantation, and brentuximab vedotin had also been administered previously. The patient received pembrolizumab and achieved complete remission after five doses. A relevant elevation of transaminases was reported after the first dose, which could not be confirmed as GvHD but was treated with steroids successfully [6]. Rigaud et al. and Hebart et al. also reported complete remission with nivulomab in ALK‐positive ALCL in two younger patients, one of them after alloHSCT, underlining the potential of this approach [4, 7]. Responses were seen even in the setting of early relapse after alloHSCT with manageable immunologic adverse events [6]. However, a Phase 1 pilot study investigating nivolumab as maintenance therapy for acute myeloid leukemia and myelodysplastic syndrome in the post‐alloHSCT setting had to be terminated after enrollment of four patients due to a higher grade unexpected toxicity. Dose regimen after alloHSCT might have to be rethought concerning the frequent treatment‐limiting toxicity (see Table 1)[8].

A lymph node specimen collected from our patient at relapse revealed no expression of PD‐L1 on tumor cells. Only a small proportion of macrophages displayed PD‐L1 expression (Figure 1). Consistent with Bennani et al., response was observed irrespective of PD‐L1 expression on tumor cells. Nevertheless, response duration is outstanding and ongoing, although treatment had to be stopped due to unacceptable potentially drug‐related toxicity [3].

In patients with non‐small cell lung cancer (NSCLC) rechallenged with nivolumab after toxicity of higher grade, Gao et al. could not identify a significant benefit in progression‐free‐survival (PFS) and overall survival (OS), but an increased risk of new adverse events, supporting a permanent discontinuation [9].

Nevertheless, concurrent with the above‐mentioned reports, especially after alloHSCT, PD‐1 blockade shows high clinical potential in selected cases even without its predefined target PD‐L1 in lymphoma tissue. Moreover, GvHD was observed and was associated with long off treatment remission. Undefined aspects of the graft‐versus‐lymphoma effect induced by nivolumab may have a higher impact than PD‐1 blockade itself (See Table 1)

**TABLE 1:** Characteristics and clinical outcomes of PD‐1 blockade in T‐cell lymphoma.

Reporting author	Disease	Prior alloHSCT	PD‐L1 status	Agent	Best response	Main adverse reactions
**Chan et al**. [6]	ALK‐negative ALCL	Yes	n.a.	Pembrolizumab	Complete remission	Hepatic
**Rigaud et al**. [4]	ALK‐positive ALCL	No	Pos.	Nivolumab	Complete remission	Not documented
**Hebart et al**. [7]	ALK‐positive ALCL	Yes	Pos.	Nivolumab	Complete remission	Pneumonitis
**Bennani et al**. [3]	Angioimmunoblastic T‐cell lymphoma, peripheral T‐cell lymphoma not otherwise specified, hepatosplenic gamma delta T‐cell lymphoma, ALK‐negative ALCL	n.a.^a^	neg.^b^	Nivolumab	Overall response rate 33%, median duration of response 3.6 months	Probable hyperprogression pancreatitis, sepsis
**Merill et al**. [5]	PTCL in first remission after autologous stem cell transplantation	No	n.a.	Pembrolizumab	18 months: 3/21 patients relapsed	Grade 3/4: respiratory failure, transaminitis, abdominal pain, colitis, diarrhea, headache

Abbreviations: ALCL, anaplastic large cell lymphoma; ALK, anaplastic lymphoma kinase; n.a., not applicable; neg., negative; PD‐L1, program death receptor‐ligand 1; pos., positive; PTCL, peripheral T‐cell lymphoma.

^a^ Only autologous stem cell transplantations were reported.

^b^ Not applicable in every patient.

In conclusion, checkpoint blockade may be an effective treatment approach in ALCL relapsing after alloHSCT, even in the absence of PD‐L1 expression on tumor cells, and new efforts for prospective trials should be made to define the maximum tolerated dose and treatment duration.

## Conflicts of Interest

Ben‐Niklas Bärmann: travel support: Kite Gilead, Medac, Incyte; membership: GLA, EBMT; speaker honoraria: Incyte; advisory role: Kite Gilead.

Kathrin Nachtkamp, Paul Jäger, Paula Kessler, Emil Novruzov, Frederik L. Giesel, Helena Peters, Maximilian Seidl, Mark Uhlenbruch, Sascha Dietrich, and Guido Kobbe declare no conflicts of interest.

## Clinical Trial Registration

The authors have confirmed clinical trial registration is not needed for this submission.

## Data Availability

n/a.

## References

[1] N. Schmitz , L. Trümper , M. Ziepert , et al., “Treatment and Prognosis of Mature T‐Cell and NK‐Cll Lymphoma: An Analysis of Patients With T‐Cell Lymphoma Treated in Studies of the German High‐Grade Non‐Hodgkin Lymphoma Study Group,” Blood 116, no. 18 (2010): 3418–3425. 20660290 10.1182/blood-2010-02-270785

[2] B. Pro , R. Advani , P. Brice , et al., “Five‐Year Results of Brentuximab Vedotin in Patients With Relapsed or Refractory Systemic Anaplastic Large Cell Lymphoma,” Blood 130, no. 25 (2017): 2709–2717. 28974506 10.1182/blood-2017-05-780049 PMC5746164

[3] N. N. Bennani , H. J. Kim , L. D. Pederson , et al., “Nivolumab in Patients With Relapsed or Refractory Peripheral T‐Cell Lymphoma: Modest Activity and Cases of Hyperprogression,” Journal for ImmunoTherapy of Cancer 10, no. 6 (2022): e004984. 35750419 10.1136/jitc-2022-004984 PMC9234908

[4] C. Rigaud , S. Abbou , V. Minard‐Colin , et al., “Efficacy of Nivolumab in a Patient With Systemic Refractory ALK+ Anaplastic Large Cell Lymphoma,” Pediatric Blood & Cancer 65, no. 4 (2018): e26902. 10.1002/pbc.26902 29193772

[5] M. H. Merrill , P. B. Dahi , R. A. Redd , et al., “A Phase 2 Study of Pembrolizumab After Autologous Stem Cell Transplantation in Patients With T‐Cell Non‐Hodgkin Lymphoma,” Blood 142, no. 7 (2023): 621–628. 37319432 10.1182/blood.2023020244 PMC10934277

[6] T. S. Chan , P. L. Khong , and Y. L. Kwong , “Pembrolizumab for Relapsed Anaplastic Large Cell Lymphoma After Allogeneic Haematopoietic Stem Cell Transplantation: Efficacy and Safety,” Annal of Hematology 95, no. 11 (2016): 1913–1915. 10.1007/s00277-016-2764-1 27473193

[7] H. Hebart , P. Lang , and W. Woessmann , “Nivolumab for Refractory Anaplastic Large Cell Lymphoma: A Case Report,” Annals of Internal Medicine 165, no. 8 (2016): 607–608. 27750310 10.7326/L16-0037

[8] A. Y. Wang , J. Kline , W. Stock , et al., “Unexpected Toxicities When Nivolumab Was Given as Maintenance Therapy Following Allogeneic Stem Cell Transplantation,” Biology of Blood and Marrow Transplantation 26, no. 5 (2020): 1025–1027. 32018063 10.1016/j.bbmt.2020.01.021

[9] M. Guo , A. M. VanderWalde , X. Yu , G. A. Vidal , and G. G. Tian , “Immune Checkpoint Inhibitor Rechallenge Safety and Efficacy in Stage IV Non‐Small Cell Lung Cancer Patients After Immune‐Related Adverse Events,” Clinical Lung Cancer 23, no. 8 (2022): 686–693. 36050243 10.1016/j.cllc.2022.07.015

